# Retraction: Transcription Regulation of HYPK by Heat Shock Factor 1

**DOI:** 10.1371/journal.pone.0360781

**Published:** 2026-10-20

**Authors:** The PLOS One Editors

Following the publication of this article [1], concerns were raised about results presented in Figs 1–4. Specifically,

- The following results appear similar, despite being used to represent different experimental conditions:
  - The left and right Fig 2D AcH4 panels.
  - The left Fig 2D RNA polymerase II panel in [1] and the Fig 2C RNA polymerase II panel in [2].
- The following panels appear to partially overlap, despite being used to represent different experimental conditions:
  - Fig 1C HYPK panel lanes 1–2 and Fig 3C HYPK panel.
  - Fig 1G HYPK panel lanes 1–2 and Fig 3G HYPK panel.
  - Fig 4C HSF1 panel lanes 2–3 and Fig 6C HYPK panel lanes 1-2.
  - Fig 4I Beta Actin panel lanes 2–3 and Fig 6G Beta Actin.

The corresponding author stated that the panel similarities were the result of figure preparation errors and stated that the original data underlying the published results are no longer available due to the time elapsed since the experiments were conducted. They offered to repeat the experiments, but PLOS does not consider that repeat experiment data are sufficient to resolve the concerns in the absence of the original data.

In light of these concerns, which call into question the integrity and reliability of the published results, the *PLOS One* Editors retract this article.

All authors responded but expressed neither agreement nor disagreement with the editorial decision.

In addition to the above concerns, PLOS is aware of additional concerns involving similarities between the right Fig 2D HSF1 panel in [1] and the right Fig 5D RNA polymerase II panel in [3], but these concerns were not investigated further.

## References

[1] Das S, Bhattacharyya NP. RETRACTED: Transcription Regulation of HYPK by Heat Shock Factor 1. PLoS One. 2014;9(1):e85552. doi: 10.1371/journal.pone.0085552 24465598 PMC3897489

[2] Das S, Bhattacharyya NP. *Trans*-activation of small EDRK-rich factor 2 (*SERF2*) promoter by Heat Shock Factor 1. Biochem Biophys Rep. 2016;7:188–94. doi: 10.1016/j.bbrep.2016.04.003 28955905 PMC5613254

[3] Das S, Bhattacharyya NP. Huntingtin interacting protein HYPK is a negative regulator of heat shock response and is downregulated in models of Huntington’s Disease. Exp Cell Res. 2016;343(2):107–17. doi: 10.1016/j.yexcr.2016.03.021 27017930

